# 5.5 MeV Electron Irradiation-Induced Transformation of Minority Carrier Traps in *p*-Type Si and Si_1−x_Ge_x_ Alloys

**DOI:** 10.3390/ma15051861

**Published:** 2022-03-02

**Authors:** Jevgenij Pavlov, Tomas Ceponis, Kornelijus Pukas, Leonid Makarenko, Eugenijus Gaubas

**Affiliations:** 1 Institute of Photonics and Nanotechnology, Vilnius University, Sauletekio Ave. 3, LT-10257 Vilnius, Lithuania; tomas.ceponis@ff.vu.lt (T.C.); kornelijus.pukas@tmi.vu.lt (K.P.); eugenijus.gaubas@ff.vu.lt (E.G.); 2 Department of Applied Mathematics and Computer Science, Belarusian State University, Independence Ave. 4, 220030 Minsk, Belarus; makleo@mail.ru

**Keywords:** electron irradiation, *p*-type silicon and silicon–germanium alloy, DLTS, acceptor removal

## Abstract

Minority carrier traps play an important role in the performance and radiation hardness of the radiation detectors operating in a harsh environment of particle accelerators, such as the up-graded sensors of the high-luminosity hadron collider (HL-HC) at CERN. It is anticipated that the sensors of the upgraded strip tracker will be based on the *p*-type silicon doped with boron. In this work, minority carrier traps in *p*-type silicon (Si) and silicon–germanium (Si_1−x_Ge_x_) alloys induced by 5.5 MeV electron irradiation were investigated by combining various modes of deep-level transient spectroscopy (DLTS) and pulsed technique of barrier evaluation using linearly increasing voltage (BELIV). These investigations were addressed to reveal the dominant radiation defects, the dopant activity transforms under local strain, as well as reactions with interstitial impurities and mechanisms of acceptor removal in *p*-type silicon (Si) and silicon–germanium (SiGe) alloys, in order to ground technological ways for radiation hardening of the advanced particle detectors. The prevailing defects of interstitial boron–oxygen (B_i_O_i_) and the vacancy–oxygen (VO) complexes, as well as the vacancy clusters, were identified using the values of activation energy reported in the literature. The activation energy shift of the radiation-induced traps with content of Ge was clarified in all the examined types of Si_1−x_Ge_x_ (with *x*= 0–0.05) materials.

## 1. Introduction

The radiation-induced boron dopant transformations in *p*-type Si lead to the so-called effect of “acceptor removal” [1,2,3,4,5], which degrades the performance of particle detectors. This happens due to the substitutional lattice site boron (B_s_) transformation into interstitial (B_i_) boron under irradiation. Further migration of this B_i_ dopant and its reaction with interstitial oxygen impurity (O_i_) in silicon crystal determines the formation of the interstitial boron–interstitial oxygen complex (B_i_O_i_). Thereby, the shallow acceptor (B_s_) in Si becomes the deep-level trap (B_i_O_i_) of minority carriers (electrons) with activation energy of about 0.25 eV. As a result, the space charge of the depleted base region and electric field in the active detector range are decreased, thereby reducing the signals of radiation detectors.

Additionally, some of radiation induced defects in Si are thermodynamically metastable [6], such as interstitial carbon–interstitial oxygen (C_i_O_i_*) complexes in *p*-type Si, as reported in our previous article [7]. The bi-stable defects can change their state under certain conditions. The metastable state can be frozen at low temperatures. The change of this state can be modified by illumination, temperature jump or applied external electrical field, where the transformations are mediated by electron–phonon interaction [6]. The B_i_O_i_ complex in Si is also a metastable defect existing in at least two configurations, namely [B_i_O_i_^A^] and [B_i_O_i_^B^] [2,3]. The density of the [B_i_O_i_^A^] configuration complexes is usually reduced under external perturbation using light or temperature (heating to 353 K) due to their transformations to [B_i_O_i_^B^] state [3]. However, the maximum concentration of [B_i_O_i_^A^] can then be restored [2,3] by retention in dark at reduced temperatures [3]. It has been reported [8,9,10] that silicon–germanium (Si_1−x_Ge_x_) alloys are promising for the production of detectors operational in radiation harsh environments. For *p*-type SiGe alloys, it is necessary to study the acceptor removal effect in boron-doped SiGe materials. Modifications of the activation energy of electron traps in Si_1−x_Ge_x,_ related to an increase in Ge content, should be clarified [1,4].

The spectrum of deep traps of majority carriers in *p*-type Si and SiGe alloys, the meta-stability of the carbon–oxygen complexes and shifts of the activation energy of these defects were investigated in our previous article [7]. In this work, the minority carrier trap spectra obtained in 5.5 MeV electron-irradiated *p*-type Si_1−x_Ge_x_ (with *x* = 0–0.05) materials were considered. The vacancy–oxygen (VO) and the boron-oxygen (B_i_O_i_) complexes, as well as vacancy clusters, were revealed to be the dominant electron traps. It was proven that boron–oxygen complexes (B_i_O_i_) of stable form [B_i_O_i_^A^] prevail in the temperature range of 65–280 K. It was shown that shifts of activation energy of the minority carrier traps appear due to an increase in Ge content in SiGe alloys.

## 2. Samples and Measurement Techniques

The Si and Si_1−x_Ge_x_ materials under consideration were grown using a Czochralski (CZ) pulling technique. The content of Ge was discretely varied in the range of *x* = 0–0.05. The alloys of Si_0.99_Ge_0.01_ and Si_0.95_Ge_0.05_ were then researched together with pure *p*-type boron-doped Si. The n^+^p structure diodes were fabricated at Scientific-Practical Materials Research Centre (SP-MRC) of Belarus National Academy of Science (NAS). All the diodes were irradiated at Laboratory of Radiation Effects in SP-MRC of Belarus NAS using a linear electron accelerator (where 283 K temperature and flux of 2 × 10^12^ cm^−2^s^−1^ were kept). The 5.5 MeV electrons were chosen according to accessible characteristics of the linear accelerator, in order to have a prevalence of point radiation defects. Different fluences (*Φ*) in the range from 5 × 10^13^ cm^−2^ to 5 × 10^14^ cm^−2^, were accumulated. Details on the irradiation regime can be found in [11].

Routine modes of the minority carrier deep-level transient spectroscopy by using electrical (MC-DLT) [7] and optical (MCT) [12,13,14,15] excess carrier injection were applied to highlight the minority carrier traps. A HERA-DLTS 1030 instrument (PhysTech GmbH) was employed to record the MC-DLT and MCT spectra using different correlation functions. The MCT regime was implemented using the continuous-wave IR light laser excitation (λ = 1064 nm) to homogeneously generate excess carriers within the depth of the sample. The IR light excitation was performed using illumination of the edge side of the diodes. The shortly illuminated samples were later kept in dark for 16 h to trace the stability of the B_i_O_i_ complexes. The DLTS spectra were recorded for the temperature range of 65–280 K. The maximum densities (*N_T_*) of radiation defects were significantly less (*N_T_* << *N_S_*) than those of dopants (*N_S_*) to have the appropriate DLTS recording regimes. The densities of the electrically active dopants were evaluated to be less than 2 × 10^15^ cm^−3^, Table 1. Thereby, a simple interaction of close defects can be assumed. The prevailing traps were identified by using the literature data defect activation energies denoted in Table 2 together with references.

A barrier evaluation technique using the linearly increasing voltage (BELIV) pulses [16] was additionally applied to clarify the role of the majority and minority carrier traps that appeared as the overlapping opposite polarity peaks within DLT spectra. The temperature- and illumination-dependent BELIV characteristics were examined. The barrier capacitance charging, the diffusion and thermal emission current components can be revealed and analyzed by recording the BELIV current transients. A closed-cycle He cryogenic system of the HERA-DLTS 1030 instrument together with diode mounting arrangement was employed to implement the BELIV measurement instrument. A pulsed generator of the linearly increasing voltage *U*(*t*)* = At* (LIV in time *t*) was connected in series with diode under tests, as well with load resistor installed within the input of a digital oscilloscope, for instance, Agilent Technologies DSO-X 5032A, to complete the electrical BELIV circuit. The same as in the DLTS experiments, continuous-wave IR light laser was employed for additional illumination of the sample.

The barrier capacitance *C_b_*(*t*) within BELIV transients depends on applied voltage pulse *U_p_*(*t*), and it varies due to charge extraction in the trap-free material. This leads to a simple relation *C_b_*(*t*) = *C_b_*_0_(1 + *U*(*t*)*/U_bi_*)^−1/2^, where barrier capacitance for a non-biased diode of an area *S* is *C_b_*_0_ = *εε*_0_*S/w*_0_ *= (εε*_0_*S*^2^*eN_D_*/2*U_bi_*)^1/2^. The symbols here represent: *ε*_0_ is a vacuum permittivity*, ε* material dielectric permittivity, *e* elementary charge, *N_D_* is the concentration of charged ions, *U_bi_* built-in potential barrier, *w*_0_ = (2*εε*_0_*U_bi_/eN_D_*)^1/2^ width of depletion for the non-biased junction, *A = U_P_/τ_PL_* is the ramp of the LIV pulse with *U_P_* peak amplitude and *τ_PL_* duration. The diffusion current determines a differential resistance of a junction, and it is expressed as *i_diff_*(*t*)* = i_diff∞_*[1* − e^-eAt/kT^*] *≅ eSn_i_*^2^*L_Dp_/N_D_τ_p_*[1* − e^-eAt/kT^*]* ≈ eSn_i_*^2^*L_Dp_/N_D_τ_p_*. Here, additional symbols represent: *kT* is thermal energy at absolute temperature *T*;* L_n,p_ *= (*D_n,p_τ_n,p_*)^1/2^ is a diffusion length for electrons (*n*) and holes (*p*) in p and n layers of a diode, respectively. *i_diff_*(*t >> kT/eA) = i_diff∞_*, *n_i_* is the intrinsic carrier density. The generation current is included within BELIV description by a modification of the volume (*w*(*t*)*S*) from which the thermally emitted carriers are collected in diode during LIV pulse evolution. The generation current *i_g_*(*t*)* = en_i_Sw*_0_(1 + *U_C_*(*t*)*/U_bi_*)^1/2^*/τ_g_* increases with voltage *U_C_*(*t*) in the rearward phase of a BELIV transient. Here, *N_D_* in expressions for *w*_0_ and *C_b_*_0_ should be replaced by their effective value *N_Def_ = N_D_ ± N_T_^±^*, due to the charged traps (*N_T_^±^* = *N_T_* − *n_T_*) of density *N_T_*. Thereby, the total current transient is described by a sum of the currents [16]:

(1) $$\begin{array}{l} i_{\Sigma }(t)=i_{C}(t)+i_{diff}(t)+i_{g}(t)=\\ \;\;=AC_{b0}\frac{1+\frac{U_{C}(t)}{2U_{bi}}}{{\left(1+\frac{U_{C}(t)}{U_{bi}}\right)}^{3/2}}+i_{diff\infty }(1-e^{-\frac{eU_{C}(t)}{kT}})+\frac{en_{i}Sw_{0}}{\tau_{g}}{\left(1+\frac{U_{C}(t)}{U_{bi}}\right)}^{1/2} \end{array}$$

The initial component of the composite current (*i*_∑_(*t*)* ≈ i_C_(t) + i_diff_*(*t*)* >> i_g_*(0)) can be exploited for evaluation of the built-in barrier *U_bi_* height. Subsequently, the carrier generation current, as well as the thermal emission lifetime, ascribed to either majority or minority carriers, can be evaluated using current value at the end of a BELIV pulse. The recorded transient usually contains the displacement and conductivity current components. The conductivity component arises within the transitional layer at the depletion boundary when the trapped carriers (*n_T_*) with steady-state concentration *n_T_*_0_ are exponentially released *n_T_*(*t*) =* n_T_*_0_exp(*−t/τ_g_*). The thermal emission lifetime [16]

(2) $$\tau_{g}\;=\;\frac{\exp(E_{T}/\mathrm{kT})}{\sigma v_{T}N_{C}}$$

is a function of several parameters: the emission cross-section *σ*, the thermal velocity *v_T_,* the density of states *N_C_* in the free carrier band and of activation (*E_T_*), as well as of thermal (*kT*) energy. The generation currents [17] from both the shallow and deep traps act simultaneously, leading to the leakage current, which is expressed as *i_g_*(*t*)* = en_i_W*(*t*)*S/<τ_g_>* through the averaged lifetime <*τ_g_*>, where the impact of shallow centers manifests within initial stages of generation current transients. The carrier emission time decreases and density of the empty capture emission centers increases with enhancement of temperature due to the change of thermal emission factor, while steady-state bias illumination saturates a filling of the carrier capture centers. The changes of material resistance *R* are dependent on the content of alloy components; irradiation parameters can also significantly modify the BELIV transients. The BELIV transient appears as a square-wave pulse in high resistivity or insulating material when barrier capacitance approaches a geometrical capacitance value. The fastest initial component of the BELIV current transient is then determined by the transition time constant *RC_b_*_0_. The linear *RC_b_*_0_ modifications can be emulated by a convolution integral [16]

(3) $$i_{RC}(t)=\frac{1}{\tau_{RC}}\int_{0}^{t}i_{C}(x)\exp[-\frac{\left(t-x\right)}{\tau_{RC}}]dx,$$

which is essential in the analysis of the initial phases of the recorded BELIV transients. There are several options for evaluation of the parameters of *C_b_*_0_, *U_bi_*, *τ_g_*, *N_Def_* and *N_T_* when using peculiar points and segments on the BELIV current transients.

**Table 2:** The electron traps revealed in the 5.5 MeV electron-irradiated Si, Si_0.99_Ge_0.01_ and Si_0.95_Ge_0.05_ diodes using MC-DLT and MCT spectra.

MC-DLT				MCT		
p-Si						
trap	*E_T_* (eV)	*N_T_* (cm^−3^)	DO	trap	*E_T_* (eV)	DO
				E1	0.150 ± 0.005	VO [5]
E2	0.240 ± 0.005	6.93 × 10^13^	B_i_O_i_	E2	0.240 ± 0.005	B_i_O_i_ [2,5,18]
				E3	0.420 ± 0.005	V_cl_ [5]
p-Si_0.99_Ge_0.01_						
trap	*E_T_* (eV)	*N_T_* (cm^−3^)	DO	trap	*E_T_* (eV)	DO
				E1	0.160 ± 0.005	VO
E2	0.240 ± 0.005	1.06 × 10^14^	B_i_O_i_	E2	0.240 ± 0.005	B_i_O_i_
				E3	0.420 ± 0.005	V_cl_
p-Si_0.95_Ge_0.05_						
				trap	*E_T_* (eV)	DO
				E1	0.240 ± 0.005	VO
				E2	0.280 ± 0.005	B_i_O_i_
				E3	0.440 ± 0.005	V_cl_

## 3. Recorded Characteristics and Extracted Parameters

To control the boron dopant concentration (*N_S_*) within the base region, the diodes composed of Si and SiGe alloy of varied Ge content were examined by measuring conventional capacitance–voltage characteristics. The reverse bias *U_R_* was increased up to 5 V. The capacitance *C* was measured using a 1 MHz frequency test signal. The dopant concentration was extracted by analyzing a slope of the *C*^−2^-*U_R_* curve. The extracted values of the dopant concentration *N_S_* in diodes composed of different Ge content material are listed in Table 1.

The close values (1.8 > *N_S_* > 1.4) × 10^15^ cm^−3^ of boron dopant concentration in diodes composed of Si and SiGe alloy with rather small Ge (≤1%) content were obtained. However, an enhancement of the Ge content within SiGe alloy to 5% leads to a clear reduction (nearly about one order of magnitude, i.e., to 1.9 × 10^14^ cm^−3^) in electrically active *B* impurities.

**Figure 1:**
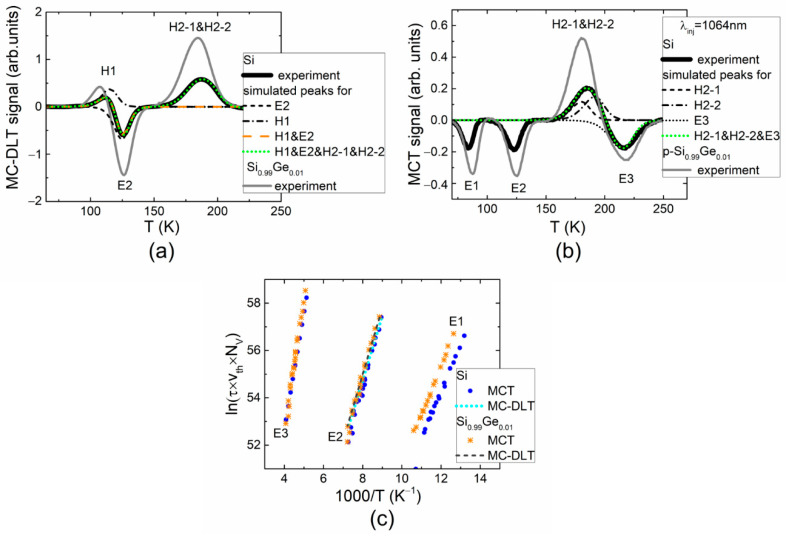
(**a**) The MC-DLTS spectra (solid curves) recorded under electrical carrier injection in 5.5 MeV electron-irradiated and boron-doped Si and Si_0.99_Ge_0.01_ diodes (the dot curve represents the simulated spectrum, including all the traps inherent for the IR illuminated diode after prolonged (16 h) retention in dark (at 293 K temperature); (**b**) The MCT spectra (solid curves) of the same Si and Si_0.99_Ge_0.01_ diodes recorded under optical injection implemented by short IR illumination of the diode edge and prolonged (16 h) retention in dark of the illuminated sample at 293 K temperature. Here, black curves represent spectra obtained for Si, while gray curves show these spectra recorded for Si_0.99_Ge_0.01_ diodes, and the dotted curves illustrate the simulated spectral peaks. (**c**) Arrhenius graphs plotted for different peaks in MC-DLT and MCT spectra. Here, *τ* denotes carrier lifetime relative to emission; *υ_th_* is the carrier thermal velocity; *N_V_* is the effective density of free carrier states.

The DLT spectra measured in electron-irradiated Si diodes under electrical (MC-DLT) and optical (MCT) carrier injection are illustrated in Figure 1a,b, respectively. The competition of the majority (H1 and H2) and minority (E1–E3) carrier traps can be deduced from the MC-DLT and MCT spectra, especially, in the range of H1 and E2 spectral peaks (within MC-DLT spectrum, Figure 1a) and H2 and E3 spectral peaks (within MCT spectrum, Figure 1b), respectively. The positive polarity spectral peaks are then assigned to the hole traps, while the negative peaks are related to the electron traps. A simulation of the overlapping spectral peaks is inevitable to highlight the defects and to extract the spectral parameters of the appropriate carrier traps. It can be deduced from Figure 1a,b that spectral positions of the H1 and E2 peaks, as well as those of the H2 and E3 traps, are corrected under simulation of counter-polarity spectral peaks. Moreover, it was clarified the overlapping spectral peaks H2-1 and H2-2 associated with majority carrier traps (Figure 1b). The peaks related to stable and metastable states of E2 trap were distinguished by short IR-illumination of the diode edge and prolonged (16 h) retention at 293 K temperature in dark of the illuminated sample. The measurements of the MCT spectra were performed, varying retention time to trace stability of traps after illumination–retention procedures. The MCT spectra were compared for the longest retention time, sufficient to stabilize the trap filling. The specified trap activation energy values were extracted using Arrhenius plots for different peaks obtained in MC-DLT and MCT spectra, as illustrated in Figure 1c.

The tentative identification of traps revealed in DLT spectra of boron-doped and electron-irradiated p-Si was performed by comparing values of activation energy extracted from Arrhenius plots and those reported in the literature, as indicated in Table 2. The hole trap H1 is assigned to the multi-vacancy (V_2_ + V_3_), while H2 trap with components H2-1 and H2-2 is related to the carbon–oxygen complex, with H2-1 being the metastable state (C_i_O_i_*) and H2-2 the stable state of this interstitial complex, respectively [7]. The minority carrier trap E1 with activation energy of 0.150 ± 0.005 eV can be associated with a vacancy–oxygen complex (VO) [5]. The electron trap E2 characterized by activation energy of 0.240 ± 0.005 eV can be ascribed to the interstitial boron–interstitial oxygen complex (B_i_O_i_) [2,5,18]. The MCT spectra recorded after IR illumination and followed retention in dark for 16 h showed that the B_i_O_i_ complex is in a stable state B_i_O_i_^A^ of maximum concentration. This defect plays a major role in the “acceptor removal” processes. Overlapping of the counter-polarity peaks, as well as optical carrier injection of carriers implemented by illumination of the diode edge, leads to difficulties in estimation of concentration of minority carrier traps. Eventually, the spectral peak E3 with activation energy of 0.420 ± 0.005 eV can be associated with a vacancy cluster (V_cl_) [5]. The parameters, such as the density (*N_T_*) of traps, their activation energy *E_T_* and defect origin (DO), are summarized in Table 2.

Nearly the same structure of spectra (relatively to those measured in p-Si) was obtained in Si_0.99_Ge_0.01_ diodes using MC-DLT and MCT modes of the DLTS recording (Figure 1a,b, gray curves). A slight shift of spectral peaks toward the higher temperature can be resolved for Si_0.99_Ge_0.01_ material diodes relatively to those recorded in p-Si. Additionally, the increased amplitudes of the spectral peaks (~*N_T_*) were obtained in Si_0.99_Ge_0.01_ diodes within both MC-DLT and MCT spectra. This result may appear due to different concentrations of electrically active boron (*N_S_*~1/*N_T_*) in Si and Si_0.99_Ge_0.01_, as indicated for *N_S_* values in Table 1. The activation energy values were extracted using Arrhenius plots, which almost coincide for *p*-type Si (Figure 1c) and Si_0.99_Ge_0.01_ diodes. It was inferred that the same radiation-induced minority carrier traps prevail within *p*-type Si and Si_0.99_Ge_0.01_ materials, as listed in Table 2.

**Figure 2:**
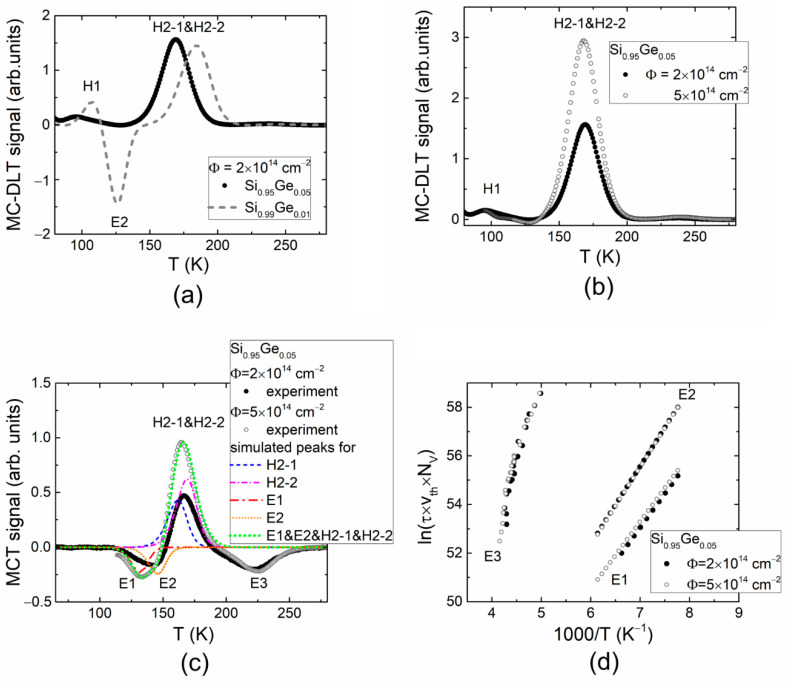
(**a**) Comparison of the MC-DLT spectra recorded by electrical injection of the excess carriers in the 2 × 10^14^ cm^−2^ fluence electron-irradiated Si_0.99_Ge_0.01_ and Si_0.95_Ge_0.05_ diodes; (**b**) Comparison of MC-DLT spectra recorded in Si_0.95_Ge_0.05_ diodes irradiated with 2 × 10^14^ cm^−2^ and 5 × 10^14^ cm^−2^ fluence where only majority carrier trap associated peaks are observed; (**c**) Comparison of the MCT spectra recorded in Si_0.95_Ge_0.05_ diodes irradiated with 2 × 10^14^ cm^−2^ and 5 × 10^14^ cm^−2^ 5.5 MeV electron fluence using optical injection of the excess carrier pairs. Here, the dotted curves illustrate the simulated spectral peaks ascribed to different traps. (**d**) Arrhenius graphs plotted for different peaks in MC-DLT and MCT spectra.

However, modifications of the MC-DLT and MCT spectra of minority carrier traps clearly depend on the fluence of electron irradiation and on content of Ge in Si_1−x_Ge_x_ diodes. A comparison of the MC-DLT spectra in 1% and 5% Ge containing Si_1−x_Ge_x_ diodes and irradiated with nearly the same electron fluence of 2 × 10^14^ e/cm^2^ is illustrated in Figure 2a. Here, no expressed minority carrier traps were obtained in Si_1−x_Ge_x_ diodes with 5% Ge, while a clear E1 peak associated with electron traps can be observed in MC-DLT spectrum recorded for Si_1−x_Ge_x_ diode with 1% Ge. Moreover, an increase in the majority carrier trap density with electron irradiation fluence can be easily deduced from Figure 2b, where the MC-DLT spectra recorded for Si_0.95_Ge_0.05_ diodes irradiated with fluences of 2 × 10^14^ cm^−2^ and 5 × 10^14^ cm^−2^ are compared. This observation can be understood assuming too small a density of electrically injected minority carriers and prevalence of the majority carrier traps of relatively large concentration. The appearance of the minority carrier traps within MCT spectra (Figure 2c) obtained using optical injection of excess carrier pairs in electron-irradiated 5% Ge containing Si_1−x_Ge_x_ diodes supports this hypothesis. There, both the minority and majority carrier trap associated DLT spectral peaks can be clearly resolved. Nevertheless, the DLTS peak position shifts with content of Ge, and irradiation fluence of Si_1−x_Ge_x_ diodes can be clearly noticed in Figure 2a,c. However, the overlapping of these peaks aggravates separation of the activation energy of the prevailing radiation defects. Spectral peak positions were partially corrected by simulating competition of traps (illustrated in Figure 2c) in the formation of the DLT signals. The Arrhenius plots of minority carrier peaks within MCT spectra of the Si_0.95_Ge_0.05_ diodes irradiated with fluences of 2 × 10^14^ cm^−2^ and 5 × 10^14^ cm^−2^ of the 5.5 MeV electrons are illustrated in Figure 2d.

Three minority-carrier-trap-related MCT spectral peaks (E1–E3, Figure 2c) were resolved for Si_0.95_Ge_0.05_ material diodes. These radiation defects can be identified using activation energy values extracted from Arrhenius plots illustrated in Figure 2d. The E1 peak with *E_T_*_,1_ ≅ 0.24 eV seems to be assigned to the vacancy–oxygen complex (VO) [5]. However, this E1 peak was not resolved within MC-DLT spectrum (Figure 2b). The E2 peak in the MCT spectrum of Si_0.95_Ge_0.05_ diode with *E_T_*_,2_ ≅ 0.28 eV seems to indicate a competition between the minority carrier traps assigned to the interstitial boron–interstitial oxygen complex (B_i_O_i_) and to either a double-charged di-vacancy (V_2_^0/=^) or vacancy clusters (V_cl_). The density of VO traps (E1) clearly increases with fluence for Ge-rich Si_0.95_Ge_0.05_ samples, as can be inferred from Figure 2c. In this case, boron (B) hardly participates in transformation of the E2 centers associated with B_i_O_i_ complex due to a reduced concentration of the electrically active boron dopants (Table 1). The E3 peak with activation energy *E_T_*_,3_ ≅ 0.44 eV, resolved within MCT spectrum of the Si_0.95_Ge_0.05_ material diode, corresponds to the vacancy clusters (V_cl_). This result was implied from consideration of the amplitudes of the minority (E3) and majority (H2) carrier trap spectral peaks and their density changes with irradiation fluence. The shifts of the minority carrier activation energy with irradiation fluence are more pronounced in Si_1−x_Ge_x_ material diodes (Figure 2c) in comparison with those values obtained for diodes composed of Si (Figure 1b).

**Figure 3:**
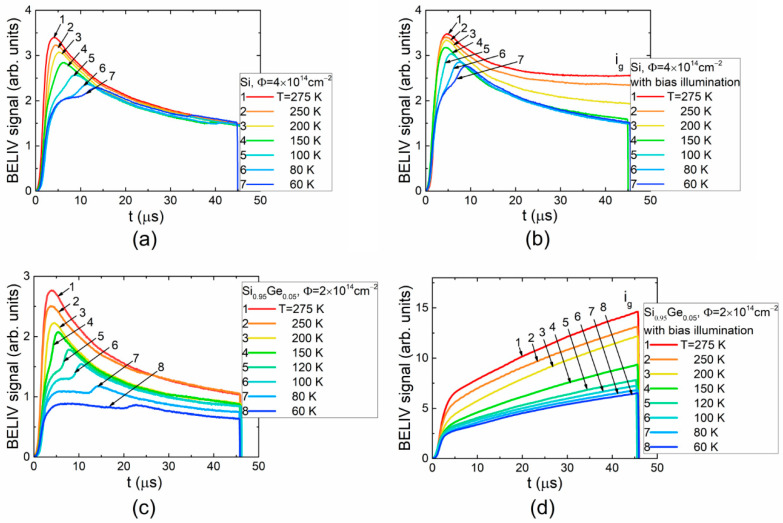
(**a**–**d**) Temperature-dependent BELIV transients in Si and Si_1−x_Ge_x_ diodes measured in dark (**a**,**c**) and under (**b**,**d**) laser illumination.

**Figure 4:**
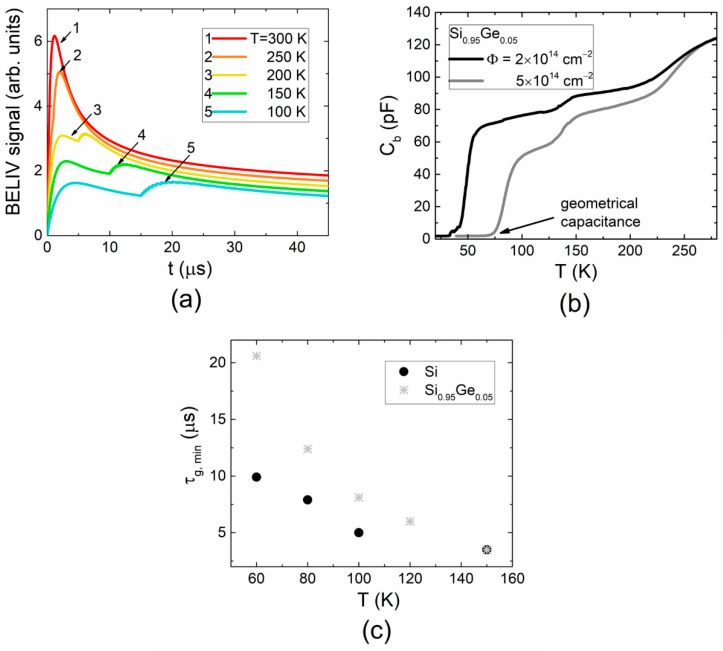
(**a**) Simulated variations (using Equations (1) and (3)) of the BELIV transients assuming changes of material resistivity, concentration of injected carriers, as well as minority and majority carrier traps of different generation lifetime. (**b**) Temperature-dependent barrier capacitance (*C_b_*) variations in diodes containing different concentration of radiation-induced traps, where *C_b_* approaches a geometrical value due to full extraction of thermally emitted carriers. (**c**) Minority carrier thermal generation lifetime as a function of temperature measured by BELIV technique in *p*-type Si and SiGe alloy.

Variations of the BELIV transient dependent on temperature and injection were examined to resolve the competition of the minority and majority carriers, as well as to clarify the role of the barrier capacitance in the formation of the DLT spectra. The recorded transients, the measured barrier capacitance temperature dependences in SiGe alloy containing different concentrations of radiation-induced traps, as well as the transients simulated through varying the parameters of carrier traps and measurement regimes (temperature, illumination), are illustrated in Figure 3 and Figure 4.

A comparison of the temperature-dependent BELIV transients, recorded in *p*-type Si (Figure 3a) and SiGe (Figure 3c) alloy diodes in dark, shows a modification of the initial stage of the BELIV transients. The delay in the peak formation within BELIV transients increases with a reduction in temperature. The appearance of the lowest initial step of the BELIV current at temperatures < 150K correlates well with temperature-dependent barrier capacitance characteristics (Figure 4a) measured using a HERA-DLTS 1030 instrument. This step is formed due to the fast capture and release of the minority carriers, which modifies the resistance of depletion and transient layers. This also determines the time- and temperature-dependent *RC_b_*_0_(*t,T*) of the diode. The rather short capture/emission of minority carriers leads to an increase in the product (*RC_b_*_0_) of the diode resistance *R* with time and a fixed value *C_b_*_0_ *≅ C_geom_* (Figure 4b) of barrier capacitance close to the geometrical (*C_geom_*) one. The duration of this initial step of the BELIV current increases with a reduction in temperature, where current amplitude is nearly invariable. This duration can be related to minority carrier thermal emission lifetime, according to Equation (2). Variation of the depleted diode resistance (*R*) increases in time *R*~1/*n* = exp(*t/τ_g,min_*)/*n_T_*_0_. The barrier capacitance restores to its value inherent for a diode, governed by the majority carrier concentration, after minority carriers are completely extracted. This instant depends on minority carrier lifetime (which increases nearly reciprocally relative to temperature) and majority carrier concentration (which decreases with temperature and depends on trap filling, modified by the external illumination), as described in [16]. Such modifications of the BELIV transient shape can be simulated by varying time- and temperature-dependent parameters of diode barrier capacitance and resistance, as illustrated in Figure 4a. Indeed, the carrier emission lifetime depends on trap parameters, activation energy and capture cross-section. The minority carrier lifetime as a function of temperature extracted for *p*-type Si and Si_0.95_Ge_0.05_ is illustrated in Figure 4c.

It can be deduced from the results of simulations illustrated in Figure 4 that minority carrier emission lifetime in SiGe alloy is significantly longer than that in Si. This result hints at the deeper trap level associated with the same species defect in SiGe alloy relative to Si. The overall reduction in BELIV current with temperature (in Figure 3a,c) simply indicates a decrease in free carrier concentration in both materials. An enhancement of the excess carrier density through the external illumination (Figure 3b,d) is followed by the BELIV current increase, which is mainly caused by the generation current term (Equation (1)). The BELIV current values at the ultimate instant of the transients approach (Figure 3b) or even prevail (Figure 3d) the BELIV peak current. The density of the minority carrier emission traps in SiGe alloys seems to be significantly larger than that of Si, as the initial step of the BELIV current is hidden (Figure 3d) by generation current term. The BELIV current values at the ultimate instant *t_p_* can be employed for comparison of the generation currents in *i_g_-T* plots. A clear prevalence of the generation current in SiGe alloys (Figure 3d) also hints that the radiation-induced minority carrier trap density is enhanced in *p*-type SiGe alloy diodes relative to that in p-Si diodes.

Electron irradiations mostly determine an introduction of point radiation defects (Table 2). It had been shown [19] that gamma irradiations of 10 kGy dose did not change significantly the conductivity of the SiGe material, while neutron irradiations sharply decreased material conductivity to 0.14% of non-irradiated material. As shown in [18], the same type of radiation defects in Si and SiGe alloys are introduced by hadron irradiations in the range of moderate energy and fluence. Nevertheless, the type inversion of n-Si had been observed [20] under Co^60^ gamma irradiations in the range (>250 Mrad) of extremely large doses. However, the appearance of the acceptor removal phenomenon and type inversion in *p*-type Si significantly depends on oxygen impurity content within pristine Si material. Therefore, the resolved VO centers within DLTS can be an indication of B acceptor removal probability in *p*-type Si and SiGe. Variations of the density of B_i_O_i_ centers and of electrically active B obtained in our research are in line with those [21] observed in p-Si irradiated with nuclear reactor neutrons over a wide range of fluences. The activation energy shifts of carrier traps, dependent on Ge content in SiGe alloys, obtained in this research, correlate rather well with such characteristics obtained in SiGe materials irradiated with electrons, protons, alpha particles and heavy ions [22,23]. However, it had been shown [24] that the production of extended defects can be suppressed in SiGe thin layers by hadron irradiations.

## 4. Discussion

**Figure 5:**
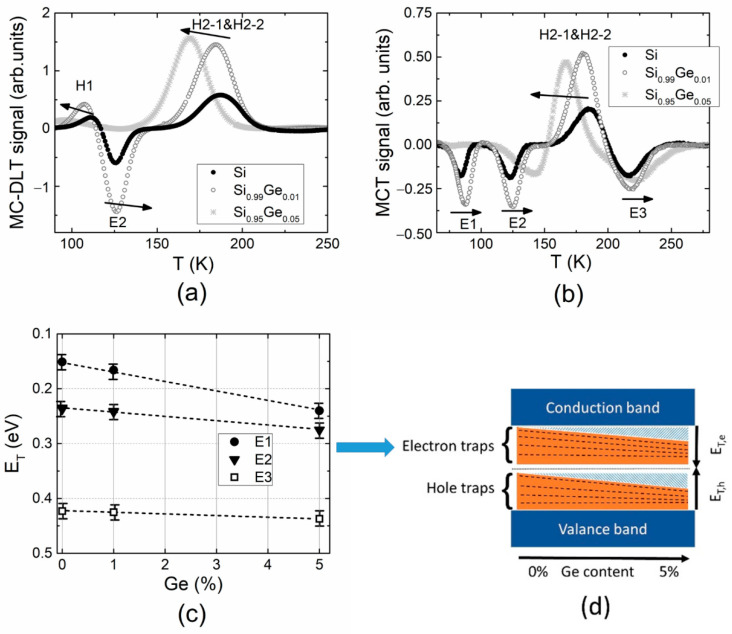
Comparison of MC-DLT (**a**) and MCT (**b**) spectra obtained in the 5.5 MeV electron-irradiated Si, Si_0.99_Ge_0.01_ and Si_0.95_Ge_0.05_ diodes; (**c**) The activation energy values (*E_T_*) of the radiation-induced traps (E1–E3) of minority carriers as a function of Ge content; (**d**) A tentative scheme of the band gap variation in *p*-type Si_1−x_Ge_x_ material and related activation energy changes in the minority carrier traps depending on the Ge content.

Variations of the activation energy values of radiation-induced traps extracted from MC-DLT (Figure 1a) and MCT (Figure 1b) spectra recorded in Si, Si_0.99_Ge_0.01_ and Si_0.95_Ge_0.05_ diodes are illustrated in Figure 5. Shifts of the DLTS peaks ascribed to majority and minority carrier trap can be inferred from these (Figure 5a,b) with the change of Ge content in SiGe alloys. The majority carrier trap ascribed peaks shift to the low-temperature wing of the DLT spectra with enhancement of Ge content within the SiGe alloy. The opposite tendency in variation of the activation energy of minority carrier traps with Ge content was revealed (Figure 5c). It was found that activation energy increases with Ge percentage. This result is supported by the BELIV characteristics, where a deeper trap level associated with the same species defect in SiGe alloy relative to that in p-Si can be inferred from Figure 4c. This shows the contrary result relative to n-type silicon–germanium alloys [25,26] where the enhancement of the majority carrier activation energy with Ge content is inherent. In *p*-type SiGe (Figure 5a), the activation energy of majority carrier traps shifts to the lower energy values with an increase in Ge content [7,27,28].

In MC-DLT spectra (Figure 5a), the peak amplitude (E2) of minority carrier traps ascribed to the interstitial boron–interstitial oxygen complex (B_i_O_i_) is rather weak for the silicon–germanium alloy with a Ge content of 5%. It has been shown [29] that Ge content modifies the density of the interstitial boron (B_i_). The boron activation energy also varies depending on local strain induced by difference in radius of the surrounding atoms. An enhancement of Ge content within SiGe compensates the local strain [29] and thereby leads to a reduction in B_i_ density. Consequently, this serves to explain a decrease in B_i_O_i_ centers and E2 peak intensity within MC-DLT spectrum (Figure 2a). On the other hand, this leads to a decrease in the electrically active dopants [30], i.e., of *N_S_*. This result proves the obtained relations among *N_S_* values in Table 1 dependent on Ge content and emulates variations of B solubility dependent on local strain. However, simultaneous action of different states [29] formed from B surrounded by Si (B-Si) atoms, and B surrounded by Si together with Ge (Si-B-Ge) atoms, stabilizes the density of the electrically active dopants (B_S_). The changes in Ge content perturb the ratio of the B-Si and Si-B-Ge states within crystal bulk and thereby local strain and density of *N_S_*. This might be a reason for the metastability of B_i_O_i_ complexes, which appeared in two configurations of [B_i_O_i_^A^] and [B_i_O_i_^B^] [2,3]. Additionally, it had been concluded [31] that most of the strain in SiGe is accommodated by variations of both the bond angle and bond length. The latter parameter also determines the changes in the formation energy of defects [32].

The indirect band gap in SiGe alloys appears between Γ- Δ valleys as it does in Si crystals, and its value as a function of Ge content is described by expression [33,34]

(4) $$E_{\mathrm{gx}}^{\Delta }(x)=1.155-0.43x+0.206x^{2}$$

In this case, the bandgap of Si_0.95_Ge_0.05_ alloy differs from that value of Si by ~0.021 eV. Diamond structure of Si_1−x_Ge_x_ still exists, as long as the Ge content is either less than 10% or more than 85%. Otherwise, SiGe will probably become a random alloy [35]. The enhancement of Ge content in SiGe alloy could also lead to the formation of an impurity band [36,37]. A tentative sketch of the bandgap in *p*-type Si_1−x_Ge_X_ alloys and arrangement of the radiation defect levels as a function of Ge content are presented in Figure 5d. Here, it is assumed that the alloy lattice still has a diamond structure. Even a small amount of substitutional Ge atoms modifies the value of energy level due to an increase in compress strain in the silicon–germanium alloy. The shifts of the activation energy of minority and majority carrier traps exhibit the opposite character, i.e., the activation energy (*E_Te_*) of the minority carrier traps increases, while the activation energy (*E_Th_*) of the majority carriers decreases due to an increase in Ge content in the SiGe alloy. This trend will probably continue until it turns into a random alloy (a polycrystalline material).

**Figure 6:**
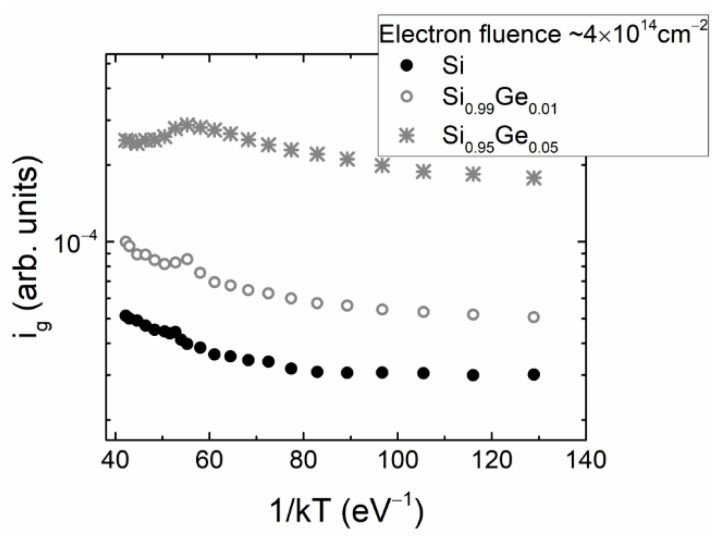
Generation current variations as a function of the inverse thermal excitation energy measured in samples under IR laser illumination.

The generation current dependence on the reciprocal thermal energy (1/*kT*) (Figure 6) extracted using BELIV current values at the ultimate instant *t_p_* (Figure 3) shows a two-componential characteristic. This implies competition between the minority and majority carrier traps within a definite range of temperatures. The elevated temperature wing (1/*kT* = 40–58) of this characteristic seems to be related to the majority carrier traps (H2), according to the temperature-dependent modifications of the BELIV transients (Figure 3a,c). The slopes of the *i_g vs._* (*kT*)^−1^ characteristics obtained for p-type Si and SiGe alloys nearly coincide for the (*kT*)^−1^ > 60 (low temperature) range. This indicates that the same minority carrier trap (probably E2) prevails there.

## 5. Summary

The prevailing defects and transformations of the minority carrier traps in 5.5 MeV electron-irradiated *p*-type Si_1−x_Ge_x_ alloys, with *x* varied in the range of 0–0.05, were examined in the temperature range of 65–280 K by the deep-level transient spectroscopy (DLTS) using electrical (MC-DLT) and optical (MCT) excess carrier injection modes. The bi-stable boron–oxygen (B_i_O_i_) complex was revealed to be the prevailing minority carrier trap in *p*-type Si_1−x_Ge_x_ alloy diodes. The relatively small density of electrically active boron–oxygen complexes in SiGe alloy with 5% of Ge content was inferred from both the MC-DLT and MCT spectra, recorded in Si_0.95_Ge_0.05_ alloy, as well as from C-V characteristics. This finding can be explained either by the fact that boron-doped Si_0.95_Ge_0.05_ diodes are more resistive to the appearance of the “acceptor removing” effect in comparison with boron-doped Si and Si_0.99_Ge_0.01_ diodes or due to Ge content modified density of the interstitial boron (B_i_). The role of the boron–oxygen (B_i_O_i_) complex is significantly reduced in Si_0.95_Ge_0.05_ alloy due to local strain. This B_i_O_i_ defect is responsible for the *B* acceptor removal and the degrading of radiation hardness. Therefore, transformations of the radiation-induced B_i_O_i_ defect in Si_0.95_Ge_0.05_ diodes can pave an advanced technology in enhancement of the detector radiation hardness based on SiGe alloys with elevated Ge content. It was also shown that the values of activation energy of radiation-induced traps of minority carriers shift to the higher values with the increase in Ge content in *p*-type SiGe alloys. The generation currents in SiGe alloys, extracted from BELIV transients, indicate that radiation-induced minority carrier trap density is enhanced in p-type SiGe alloy diodes relative to that in p-Si diodes.

## Figures and Tables

**Table 1:** The boron dopant concentration in diodes composed of Si and SiGe alloy evaluated using C-V characteristics.

Sample	*N_S_* (cm^−3^)
Si	1.8 × 10^15^
Si_0.99_Ge_0.01_	1.4 × 10^15^
Si_0.95_Ge_0.05_	1.9 × 10^14^

## Data Availability

Some of the data presented in this study are available on request from the corresponding author.
